# Supplementary material for: Individual retrotransposon integrants are differentially controlled by KZFP/KAP1-dependent histone methylation, DNA methylation and TET-mediated hydroxymethylation in naïve embryonic stem cells
Source: Epigenetics Chromatin. 2018 Feb 26;11:7. doi: 10.1186/s13072-018-0177-1 (PMC6389204; doi:10.1186/s13072-018-0177-1)
Supplement: Supplementary file 11 — Additional file 11. Pattern analysis. [file 13072_2018_177_MOESM11_ESM.zip › Patterns analysis/tables/p21_2_repNameOverr.html]

p21\_2 . RepName overrepresentation (expressed EREs as background)


# p21\_2 . RepName overrepresentation (expressed EREs as background)

repName | Expressed | inPattern | inPatternProp | pval |
| repName | Expressed | inPattern | inPatternProp | pval |
| --- | --- | --- | --- | --- |
| IAPEz-int | 1540 | 0 | 0.0 | 1 |
| L1Md\_T | 1164 | 0 | 0.0 | 1 |
| MurSatRep1 | 937 | 0 | 0.0 | 1 |
| MMSAT4 | 871 | 1 | 0.1 | 1 |
| B3 | 744 | 0 | 0.0 | 1 |
| MERVL-int | 719 | 0 | 0.0 | 1 |
| ID\_B1 | 687 | 0 | 0.0 | 1 |
| L1Md\_A | 636 | 0 | 0.0 | 1 |
| AT\_rich | 616 | 0 | 0.0 | 1 |
| RSINE1 | 481 | 0 | 0.0 | 1 |
| B1\_Mus1 | 464 | 0 | 0.0 | 1 |
| PB1D10 | 454 | 0 | 0.0 | 1 |
| B2\_Mm2 | 424 | 1 | 0.2 | 1 |
| L1Md\_F2 | 390 | 0 | 0.0 | 1 |
| B3A | 377 | 0 | 0.0 | 1 |
| (TG)n | 375 | 0 | 0.0 | 1 |
| B4A | 346 | 1 | 0.3 | 1 |
| B2\_Mm1a | 340 | 0 | 0.0 | 1 |
| B1\_Mus2 | 322 | 0 | 0.0 | 1 |
| MMETn-int | 301 | 0 | 0.0 | 1 |
| MMERVK10C-int | 284 | 0 | 0.0 | 1 |
| PB1D9 | 283 | 0 | 0.0 | 1 |
| B1F | 275 | 0 | 0.0 | 1 |
| (CA)n | 235 | 0 | 0.0 | 1 |
| B2\_Mm1t | 224 | 0 | 0.0 | 1 |
| ETnERV-int | 223 | 0 | 0.0 | 1 |
| B4 | 220 | 0 | 0.0 | 1 |
| B1\_Mur1 | 212 | 0 | 0.0 | 1 |
| GC\_rich | 205 | 0 | 0.0 | 1 |
| L1\_Mus1 | 200 | 0 | 0.0 | 1 |
| B1\_Mur4 | 196 | 0 | 0.0 | 1 |
| B1\_Mur2 | 195 | 0 | 0.0 | 1 |
| MT2\_Mm | 193 | 0 | 0.0 | 1 |
| GA-rich | 187 | 0 | 0.0 | 1 |
| PB1D7 | 185 | 0 | 0.0 | 1 |
| T-rich | 182 | 0 | 0.0 | 1 |
| L1\_Mus3 | 178 | 0 | 0.0 | 1 |
| (T)n | 158 | 1 | 0.6 | 1 |
| ORR1A3-int | 154 | 1 | 0.6 | 1 |
| MIRb | 149 | 0 | 0.0 | 1 |
| B1\_Mur3 | 139 | 0 | 0.0 | 1 |
| B1\_Mm | 135 | 0 | 0.0 | 1 |
| MMERVK9C\_I-int | 133 | 0 | 0.0 | 1 |
| MTD | 127 | 1 | 0.8 | 1 |
| ID4 | 125 | 0 | 0.0 | 1 |
| (TTTTG)n | 119 | 0 | 0.0 | 1 |
| CT-rich | 117 | 0 | 0.0 | 1 |
| ID4\_ | 112 | 0 | 0.0 | 1 |
| B1F1 | 111 | 0 | 0.0 | 1 |
| MIR | 111 | 0 | 0.0 | 1 |
| MIRc | 110 | 0 | 0.0 | 1 |
| C-rich | 109 | 0 | 0.0 | 1 |
| (A)n | 108 | 0 | 0.0 | 1 |
| MTA\_Mm-int | 107 | 0 | 0.0 | 1 |
| MT2B | 106 | 0 | 0.0 | 1 |
| B1F2 | 102 | 0 | 0.0 | 1 |
| (TTTG)n | 97 | 0 | 0.0 | 1 |
| (TC)n | 96 | 1 | 1.0 | 1 |
| L1Md\_F3 | 95 | 0 | 0.0 | 1 |
| MMERVK9E\_I-int | 95 | 0 | 0.0 | 1 |
| PB1 | 95 | 0 | 0.0 | 1 |
| (CAG)n | 93 | 0 | 0.0 | 1 |
| ORR1A0-int | 93 | 0 | 0.0 | 1 |
| L1\_Mus2 | 88 | 0 | 0.0 | 1 |
| Lx | 88 | 0 | 0.0 | 1 |
| RLTR45-int | 88 | 0 | 0.0 | 1 |
| Lx8 | 87 | 0 | 0.0 | 1 |
| (GGA)n | 83 | 0 | 0.0 | 1 |
| IAPEY3-int | 82 | 0 | 0.0 | 1 |
| Lx7 | 82 | 0 | 0.0 | 1 |
| A-rich | 80 | 0 | 0.0 | 1 |
| URR1B | 77 | 1 | 1.3 | 1 |
| L2a | 75 | 0 | 0.0 | 1 |
| Lx9 | 75 | 0 | 0.0 | 1 |
| MIR3 | 75 | 0 | 0.0 | 1 |
| ETnERV2-int | 72 | 0 | 0.0 | 1 |
| MERVL\_2A-int | 72 | 0 | 0.0 | 1 |
| L2 | 71 | 0 | 0.0 | 1 |
| RLTR10-int | 70 | 0 | 0.0 | 1 |
| RMER16-int | 70 | 0 | 0.0 | 1 |
| Lx6 | 69 | 0 | 0.0 | 1 |
| RLTR1B-int | 68 | 0 | 0.0 | 1 |
| MMERGLN-int | 64 | 0 | 0.0 | 1 |
| MTC | 64 | 0 | 0.0 | 1 |
| URR1A | 64 | 0 | 0.0 | 1 |
| (CGG)n | 63 | 0 | 0.0 | 1 |
| L2c | 62 | 0 | 0.0 | 1 |
| ORR1A2 | 61 | 0 | 0.0 | 1 |
| RMER15 | 61 | 0 | 0.0 | 1 |
| L3 | 60 | 0 | 0.0 | 1 |
| ORR1B1 | 60 | 0 | 0.0 | 1 |
| IAPEy-int | 59 | 0 | 0.0 | 1 |
| Lx5 | 59 | 0 | 0.0 | 1 |
| (TA)n | 57 | 0 | 0.0 | 1 |
| (CAAA)n | 56 | 0 | 0.0 | 1 |
| LSU-rRNA\_Hsa | 56 | 0 | 0.0 | 1 |
| L2b | 55 | 0 | 0.0 | 1 |
| Lx2B | 55 | 0 | 0.0 | 1 |
| ORR1A2-int | 55 | 0 | 0.0 | 1 |
| L1Md\_Gf | 54 | 0 | 0.0 | 1 |
| ORR1A1-int | 53 | 0 | 0.0 | 1 |
| ORR1A4-int | 53 | 0 | 0.0 | 1 |
| (TTTTTG)n | 52 | 0 | 0.0 | 1 |
| L1\_Mur2 | 49 | 0 | 0.0 | 1 |
| (CAAAA)n | 48 | 0 | 0.0 | 1 |
| (GA)n | 48 | 0 | 0.0 | 1 |
| Lx2 | 48 | 0 | 0.0 | 1 |
| MT2A | 46 | 0 | 0.0 | 1 |
| L1\_Mus4 | 45 | 0 | 0.0 | 1 |
| IAPEY4\_I-int | 44 | 0 | 0.0 | 1 |
| Lx8b | 44 | 0 | 0.0 | 1 |
| MMVL30-int | 44 | 0 | 0.0 | 1 |
| MT2B2 | 44 | 0 | 0.0 | 1 |
| MTA\_Mm | 44 | 0 | 0.0 | 1 |
| ORR1E | 44 | 0 | 0.0 | 1 |
| (CCG)n | 43 | 0 | 0.0 | 1 |
| L1\_Mur3 | 43 | 0 | 0.0 | 1 |
| Lx3\_Mus | 43 | 0 | 0.0 | 1 |
| ORR1B1-int | 43 | 0 | 0.0 | 1 |
| L1\_Mm | 42 | 0 | 0.0 | 1 |
| MTE2a | 42 | 0 | 0.0 | 1 |
| IAP-d-int | 41 | 0 | 0.0 | 1 |
| ORR1D1 | 41 | 0 | 0.0 | 1 |
| RMER19B | 41 | 0 | 0.0 | 1 |
| MER5A | 40 | 0 | 0.0 | 1 |
| (TTTC)n | 40 | 0 | 0.0 | 1 |
| MTEa | 39 | 0 | 0.0 | 1 |
| L1VL2 | 38 | 0 | 0.0 | 1 |
| MTE2b | 38 | 0 | 0.0 | 1 |
| (CTG)n | 37 | 0 | 0.0 | 1 |
| ETnERV3-int | 37 | 0 | 0.0 | 1 |
| RLTR6-int | 37 | 0 | 0.0 | 1 |
| RLTR9E | 37 | 0 | 0.0 | 1 |
| (CCA)n | 36 | 0 | 0.0 | 1 |
| L1M2 | 36 | 0 | 0.0 | 1 |
| RLTR17B\_Mm | 36 | 0 | 0.0 | 1 |
| (ATG)n | 35 | 0 | 0.0 | 1 |
| L1M5 | 35 | 0 | 0.0 | 1 |
| (TTG)n | 35 | 0 | 0.0 | 1 |
| L1Md\_F | 34 | 0 | 0.0 | 1 |
| MTC-int | 34 | 0 | 0.0 | 1 |
| RMER6A | 34 | 0 | 0.0 | 1 |
| G-rich | 33 | 0 | 0.0 | 1 |
| ORR1D2 | 33 | 0 | 0.0 | 1 |
| (TCC)n | 32 | 0 | 0.0 | 1 |
| ERVB4\_2-I\_MM-int | 31 | 0 | 0.0 | 1 |
| L1M4 | 31 | 0 | 0.0 | 1 |
| MTD-int | 31 | 0 | 0.0 | 1 |
| ORR1C2 | 31 | 0 | 0.0 | 1 |
| RLTR19-int | 31 | 0 | 0.0 | 1 |
| RLTR4\_MM-int | 31 | 0 | 0.0 | 1 |
| Lx3C | 30 | 0 | 0.0 | 1 |
| L4 | 29 | 0 | 0.0 | 1 |
| BC1\_Mm | 28 | 0 | 0.0 | 1 |
| Lx4A | 28 | 0 | 0.0 | 1 |
| MER2 | 28 | 0 | 0.0 | 1 |
| RMER1B | 28 | 0 | 0.0 | 1 |
| (CCCCAG)n | 27 | 0 | 0.0 | 1 |
| L1VL4 | 27 | 0 | 0.0 | 1 |
| MTB | 27 | 0 | 0.0 | 1 |
| ORR1A4 | 27 | 0 | 0.0 | 1 |
| ERVB4\_1B-I\_MM-int | 26 | 0 | 0.0 | 1 |
| MTEb | 25 | 0 | 0.0 | 1 |
| MTE-int | 25 | 0 | 0.0 | 1 |
| RLTR20C1\_MM | 25 | 0 | 0.0 | 1 |
| IAPA\_MM-int | 24 | 0 | 0.0 | 1 |
| ID | 24 | 0 | 0.0 | 1 |
| MuRRS4-int | 24 | 0 | 0.0 | 1 |
| RMER10A | 24 | 0 | 0.0 | 1 |
| (CAAAAA)n | 23 | 0 | 0.0 | 1 |
| ID2 | 23 | 0 | 0.0 | 1 |
| RMER5 | 23 | 0 | 0.0 | 1 |
| MMERVK10D3\_I-int | 22 | 0 | 0.0 | 1 |
| MYSERV6-int | 22 | 0 | 0.0 | 1 |
| ORR1C1 | 22 | 0 | 0.0 | 1 |
| RLTR17 | 22 | 0 | 0.0 | 1 |
| RLTR25B | 22 | 0 | 0.0 | 1 |
| RLTR44-int | 22 | 0 | 0.0 | 1 |
| ZP3AR | 22 | 0 | 0.0 | 1 |
| (GAA)n | 21 | 0 | 0.0 | 1 |
| Lx3A | 21 | 0 | 0.0 | 1 |
| Lx3B | 21 | 0 | 0.0 | 1 |
| MER20 | 21 | 0 | 0.0 | 1 |
| MERV1\_I-int | 21 | 0 | 0.0 | 1 |
| MLT1D | 21 | 0 | 0.0 | 1 |
| MYSERV16\_I-int | 21 | 0 | 0.0 | 1 |
| IAPLTR3-int | 20 | 0 | 0.0 | 1 |
| L1MC4 | 20 | 0 | 0.0 | 1 |
| MT2C\_Mm | 20 | 0 | 0.0 | 1 |
| RMER17C | 20 | 0 | 0.0 | 1 |
| RMER4B | 20 | 0 | 0.0 | 1 |
| (TATG)n | 20 | 0 | 0.0 | 1 |
| (TTCC)n | 20 | 0 | 0.0 | 1 |
| IAP1-MM\_I-int | 19 | 0 | 0.0 | 1 |
| RLTR15 | 19 | 0 | 0.0 | 1 |
| RodERV21-int | 19 | 0 | 0.0 | 1 |
| (CAA)n | 18 | 0 | 0.0 | 1 |
| MLT1B | 18 | 0 | 0.0 | 1 |
| MLT1C | 18 | 0 | 0.0 | 1 |
| MurERV4-int | 18 | 0 | 0.0 | 1 |
| ORR1D2-int | 18 | 0 | 0.0 | 1 |
| RCHARR1 | 18 | 0 | 0.0 | 1 |
| RMER12 | 18 | 0 | 0.0 | 1 |
| RMER17C-int | 18 | 0 | 0.0 | 1 |
| RMER19C | 18 | 0 | 0.0 | 1 |
| RMER3D-int | 18 | 0 | 0.0 | 1 |
| (TCTG)n | 18 | 0 | 0.0 | 1 |
| LTRIS2 | 17 | 0 | 0.0 | 1 |
| Lx4B | 17 | 0 | 0.0 | 1 |
| MusHAL1 | 17 | 0 | 0.0 | 1 |
| L1MB7 | 16 | 0 | 0.0 | 1 |
| L1MB8 | 16 | 0 | 0.0 | 1 |
| L1\_Mur1 | 16 | 0 | 0.0 | 1 |
| MER5B | 16 | 0 | 0.0 | 1 |
| ORR1B2 | 16 | 0 | 0.0 | 1 |
| RLTR21 | 16 | 0 | 0.0 | 1 |
| RMER1A | 16 | 0 | 0.0 | 1 |
| RMER30 | 16 | 1 | 6.2 | 1 |
| Tigger7 | 16 | 0 | 0.0 | 1 |
| (TTTTC)n | 16 | 0 | 0.0 | 1 |
| (CATA)n | 15 | 0 | 0.0 | 1 |
| ERVB2\_1A-I\_MM-int | 15 | 0 | 0.0 | 1 |
| L1MA4 | 15 | 0 | 0.0 | 1 |
| L1\_Rod | 15 | 0 | 0.0 | 1 |
| L1VL1 | 15 | 0 | 0.0 | 1 |
| MuLV-int | 15 | 0 | 0.0 | 1 |
| ORR1E-int | 15 | 0 | 0.0 | 1 |
| RLTR1B | 15 | 0 | 0.0 | 1 |
| (CAGAGA)n | 14 | 0 | 0.0 | 1 |
| MTB\_Mm | 14 | 0 | 0.0 | 1 |
| RLTR11A2 | 14 | 1 | 7.1 | 1 |
| RLTRETN\_Mm | 14 | 0 | 0.0 | 1 |
| RMER15-int | 14 | 0 | 0.0 | 1 |
| (TCTCTG)n | 14 | 0 | 0.0 | 1 |
| (TTA)n | 14 | 0 | 0.0 | 1 |
| L1M3 | 13 | 0 | 0.0 | 1 |
| L1MA6 | 13 | 0 | 0.0 | 1 |
| L1MC | 13 | 0 | 0.0 | 1 |
| LTRIS4A | 13 | 0 | 0.0 | 1 |
| LTRIS\_Mus | 13 | 0 | 0.0 | 1 |
| MuRRS-int | 13 | 0 | 0.0 | 1 |
| ORR1D-int | 13 | 0 | 0.0 | 1 |
| RLTR20B1 | 13 | 0 | 0.0 | 1 |
| RLTR42-int | 13 | 0 | 0.0 | 1 |
| Tigger1 | 13 | 0 | 0.0 | 1 |
| YREP\_Mm | 13 | 0 | 0.0 | 1 |
| 4.5SRNA | 12 | 0 | 0.0 | 1 |
| (CAT)n | 12 | 0 | 0.0 | 1 |
| IAPLTR2\_Mm | 12 | 0 | 0.0 | 1 |
| L1MB5 | 12 | 0 | 0.0 | 1 |
| L1MEg | 12 | 0 | 0.0 | 1 |
| Lx10 | 12 | 0 | 0.0 | 1 |
| MT-int | 12 | 0 | 0.0 | 1 |
| MYSERV-int | 12 | 0 | 0.0 | 1 |
| RLTR13E | 12 | 0 | 0.0 | 1 |
| RLTR18-int | 12 | 0 | 0.0 | 1 |
| RLTR28 | 12 | 0 | 0.0 | 1 |
| RMER6B | 12 | 0 | 0.0 | 1 |
| RMER6C | 12 | 0 | 0.0 | 1 |
| (TGG)n | 12 | 0 | 0.0 | 1 |
| tRNA-Ala-GCY\_ | 12 | 0 | 0.0 | 1 |
| (CAGA)n | 11 | 0 | 0.0 | 1 |
| HAL1 | 11 | 0 | 0.0 | 1 |
| L1M4c | 11 | 0 | 0.0 | 1 |
| L1MD | 11 | 0 | 0.0 | 1 |
| MER58B | 11 | 0 | 0.0 | 1 |
| MLT1K | 11 | 0 | 0.0 | 1 |
| MLTR25A | 11 | 0 | 0.0 | 1 |
| ORR1A3 | 11 | 0 | 0.0 | 1 |
| RLTR22\_Mur | 11 | 0 | 0.0 | 1 |
| RLTR25A | 11 | 0 | 0.0 | 1 |
| RLTR9D | 11 | 0 | 0.0 | 1 |
| RMER16 | 11 | 0 | 0.0 | 1 |
| Tigger5 | 11 | 0 | 0.0 | 1 |
| AmnSINE1 | 10 | 0 | 0.0 | 1 |
| BGLII | 10 | 0 | 0.0 | 1 |
| Charlie1a | 10 | 0 | 0.0 | 1 |
| IAPLTR1\_Mm | 10 | 0 | 0.0 | 1 |
| L1MB3 | 10 | 0 | 0.0 | 1 |
| L1MC1 | 10 | 0 | 0.0 | 1 |
| L1MC4a | 10 | 0 | 0.0 | 1 |
| MER3 | 10 | 0 | 0.0 | 1 |
| MLT1A0 | 10 | 0 | 0.0 | 1 |
| MTB-int | 10 | 0 | 0.0 | 1 |
| ORR1A0 | 10 | 0 | 0.0 | 1 |
| ORR1A1 | 10 | 0 | 0.0 | 1 |
| ORR1D1-int | 10 | 0 | 0.0 | 1 |
| RLTR13G | 10 | 0 | 0.0 | 1 |
| RLTR6B\_Mm | 10 | 0 | 0.0 | 1 |
| RMER10B | 10 | 0 | 0.0 | 1 |
| (TAGA)n | 10 | 0 | 0.0 | 1 |
| (TATATG)n | 10 | 0 | 0.0 | 1 |
| (TCTA)n | 10 | 0 | 0.0 | 1 |
| (TTTA)n | 10 | 0 | 0.0 | 1 |
| (CGTG)n | 9 | 0 | 0.0 | 1 |
| (GTTTG)n | 9 | 0 | 0.0 | 1 |
| IAPLTR2a | 9 | 0 | 0.0 | 1 |
| L1MDa | 9 | 0 | 0.0 | 1 |
| L1ME1 | 9 | 0 | 0.0 | 1 |
| MER21B | 9 | 0 | 0.0 | 1 |
| MER33 | 9 | 0 | 0.0 | 1 |
| MER5A1 | 9 | 0 | 0.0 | 1 |
| MERVK26-int | 9 | 0 | 0.0 | 1 |
| MLT1A1 | 9 | 0 | 0.0 | 1 |
| RLTR11A | 9 | 0 | 0.0 | 1 |
| RLTR20C2\_MM | 9 | 0 | 0.0 | 1 |
| RLTR33 | 9 | 0 | 0.0 | 1 |
| RMER19A | 9 | 0 | 0.0 | 1 |
| RMER2 | 9 | 0 | 0.0 | 1 |
| RMER4A | 9 | 0 | 0.0 | 1 |
| (TCCA)n | 9 | 0 | 0.0 | 1 |
| (TTTTA)n | 9 | 0 | 0.0 | 1 |
| 5S | 8 | 0 | 0.0 | 1 |
| Charlie8 | 8 | 0 | 0.0 | 1 |
| HAL1-3A\_ME | 8 | 0 | 0.0 | 1 |
| L1MA5 | 8 | 0 | 0.0 | 1 |
| L1MC5 | 8 | 0 | 0.0 | 1 |
| MER58A | 8 | 0 | 0.0 | 1 |
| MERV1\_LTR | 8 | 0 | 0.0 | 1 |
| MURVY-int | 8 | 0 | 0.0 | 1 |
| ORR1C2-int | 8 | 0 | 0.0 | 1 |
| polypurine | 8 | 0 | 0.0 | 1 |
| RLTR13D3A1 | 8 | 0 | 0.0 | 1 |
| RLTR30D2\_MM | 8 | 0 | 0.0 | 1 |
| RLTR45 | 8 | 0 | 0.0 | 1 |
| RMER17A | 8 | 0 | 0.0 | 1 |
| RMER17B | 8 | 0 | 0.0 | 1 |
| RMER1C | 8 | 0 | 0.0 | 1 |
| RMER20B | 8 | 0 | 0.0 | 1 |
| SSU-rRNA\_Hsa | 8 | 0 | 0.0 | 1 |
| (TAAA)n | 8 | 0 | 0.0 | 1 |
| BGLII\_B | 7 | 0 | 0.0 | 1 |
| Charlie4z | 7 | 0 | 0.0 | 1 |
| ERVB4\_1B-LTR\_MM | 7 | 0 | 0.0 | 1 |
| ERVL-B4-int | 7 | 0 | 0.0 | 1 |
| (GAAA)n | 7 | 0 | 0.0 | 1 |
| HAL1-2a\_MD | 7 | 0 | 0.0 | 1 |
| L1ME2 | 7 | 0 | 0.0 | 1 |
| LFSINE\_Vert | 7 | 0 | 0.0 | 1 |
| MARNA | 7 | 0 | 0.0 | 1 |
| MER21C | 7 | 0 | 0.0 | 1 |
| MLT1L | 7 | 0 | 0.0 | 1 |
| MurERV4\_19-int | 7 | 0 | 0.0 | 1 |
| polypyrimidine | 7 | 0 | 0.0 | 1 |
| RLTR10D | 7 | 0 | 0.0 | 1 |
| RLTR11C\_MM | 7 | 0 | 0.0 | 1 |
| RLTR12C | 7 | 0 | 0.0 | 1 |
| RLTR14-int | 7 | 0 | 0.0 | 1 |
| RLTR19 | 7 | 0 | 0.0 | 1 |
| RLTR40 | 7 | 0 | 0.0 | 1 |
| RLTR49 | 7 | 0 | 0.0 | 1 |
| RMER17A2 | 7 | 0 | 0.0 | 1 |
| RMER17D2 | 7 | 0 | 0.0 | 1 |
| RMER21A | 7 | 0 | 0.0 | 1 |
| (TCCC)n | 7 | 0 | 0.0 | 1 |
| (TGGA)n | 7 | 0 | 0.0 | 1 |
| Tigger10 | 7 | 0 | 0.0 | 1 |
| tRNA-Ala-GCA | 7 | 0 | 0.0 | 1 |
| U6 | 7 | 0 | 0.0 | 1 |
| 7SLRNA | 6 | 0 | 0.0 | 1 |
| (CCCCCA)n | 6 | 0 | 0.0 | 1 |
| (CGGGG)n | 6 | 0 | 0.0 | 1 |
| Charlie1 | 6 | 0 | 0.0 | 1 |
| ERVB7\_1-LTR\_MM | 6 | 0 | 0.0 | 1 |
| ERVB7\_2B-LTR\_MM | 6 | 0 | 0.0 | 1 |
| (GGAGAA)n | 6 | 0 | 0.0 | 1 |
| GSAT\_MM | 6 | 0 | 0.0 | 1 |
| L1M4b | 6 | 0 | 0.0 | 1 |
| L1ME4a | 6 | 0 | 0.0 | 1 |
| L1MEc | 6 | 0 | 0.0 | 1 |
| L3b | 6 | 0 | 0.0 | 1 |
| LTRIS\_Mm | 6 | 0 | 0.0 | 1 |
| MER44B | 6 | 0 | 0.0 | 1 |
| MER89 | 6 | 0 | 0.0 | 1 |
| MLT1J | 6 | 0 | 0.0 | 1 |
| MLT2F | 6 | 0 | 0.0 | 1 |
| MLTR18B\_MM | 6 | 0 | 0.0 | 1 |
| MLTR31F\_MM | 6 | 0 | 0.0 | 1 |
| MTE2b-int | 6 | 0 | 0.0 | 1 |
| ORR1B2-int | 6 | 0 | 0.0 | 1 |
| ORR1C1-int | 6 | 0 | 0.0 | 1 |
| RLTR10C | 6 | 0 | 0.0 | 1 |
| RLTR11B | 6 | 0 | 0.0 | 1 |
| RLTR12D | 6 | 0 | 0.0 | 1 |
| RLTR13B2 | 6 | 0 | 0.0 | 1 |
| RLTR13D2 | 6 | 0 | 0.0 | 1 |
| RLTR18B | 6 | 0 | 0.0 | 1 |
| RLTR41 | 6 | 0 | 0.0 | 1 |
| RLTR50B | 6 | 0 | 0.0 | 1 |
| RMER13B | 6 | 0 | 0.0 | 1 |
| RMER6-int | 6 | 0 | 0.0 | 1 |
| SRV\_MM-int | 6 | 0 | 0.0 | 1 |
| Tigger15a | 6 | 0 | 0.0 | 1 |
| (TTCTC)n | 6 | 0 | 0.0 | 1 |
| AmnSINE2 | 5 | 0 | 0.0 | 1 |
| (ATTG)n | 5 | 0 | 0.0 | 1 |
| (CAAAC)n | 5 | 0 | 0.0 | 1 |
| (CACCC)n | 5 | 0 | 0.0 | 1 |
| (CCAA)n | 5 | 0 | 0.0 | 1 |
| Cheshire | 5 | 0 | 0.0 | 1 |
| (CTCA)n | 5 | 0 | 0.0 | 1 |
| (CTGGGG)n | 5 | 0 | 0.0 | 1 |
| CYRA11\_Mm | 5 | 1 | 20.0 | 1 |
| ERVB4\_1-I\_MM-int | 5 | 0 | 0.0 | 1 |
| (GCCTG)n | 5 | 0 | 0.0 | 1 |
| L1MC3 | 5 | 0 | 0.0 | 1 |
| L1MCa | 5 | 0 | 0.0 | 1 |
| L1MEf | 5 | 0 | 0.0 | 1 |
| Lx2A | 5 | 0 | 0.0 | 1 |
| MamGyp-int | 5 | 0 | 0.0 | 1 |
| MER53 | 5 | 0 | 0.0 | 1 |
| MLT2B1 | 5 | 0 | 0.0 | 1 |
| MLT2D | 5 | 0 | 0.0 | 1 |
| MTB\_Mm-int | 5 | 0 | 0.0 | 1 |
| RLTR13A1 | 5 | 0 | 0.0 | 1 |
| RLTR13D6 | 5 | 0 | 0.0 | 1 |
| RLTR14 | 5 | 0 | 0.0 | 1 |
| RLTR22\_Mus | 5 | 0 | 0.0 | 1 |
| RLTR31C\_MM | 5 | 0 | 0.0 | 1 |
| RLTR48A | 5 | 0 | 0.0 | 1 |
| RLTR9A | 5 | 0 | 0.0 | 1 |
| RMER12B | 5 | 0 | 0.0 | 1 |
| RMER17D | 5 | 0 | 0.0 | 1 |
| (TAA)n | 5 | 0 | 0.0 | 1 |
| (TGAA)n | 5 | 0 | 0.0 | 1 |
| Tigger6a | 5 | 0 | 0.0 | 1 |
| (TTC)n | 5 | 0 | 0.0 | 1 |
| (TTGG)n | 5 | 0 | 0.0 | 1 |
| (TTTCC)n | 5 | 0 | 0.0 | 1 |
| (TTTTTA)n | 5 | 0 | 0.0 | 1 |
| 7SK | 4 | 0 | 0.0 | 1 |
| BGLII\_C | 4 | 0 | 0.0 | 1 |
| (CCCCCT)n | 4 | 0 | 0.0 | 1 |
| (CCCCG)n | 4 | 0 | 0.0 | 1 |
| (CCTG)n | 4 | 0 | 0.0 | 1 |
| (CGA)n | 4 | 0 | 0.0 | 1 |
| Charlie12 | 4 | 0 | 0.0 | 1 |
| Charlie1b | 4 | 0 | 0.0 | 1 |
| Charlie5 | 4 | 0 | 0.0 | 1 |
| ERVB2\_1-I\_MM-int | 4 | 0 | 0.0 | 1 |
| IAPLTR2b | 4 | 0 | 0.0 | 1 |
| IAPLTR4\_I | 4 | 0 | 0.0 | 1 |
| L1MA7 | 4 | 0 | 0.0 | 1 |
| L1MA8 | 4 | 0 | 0.0 | 1 |
| L1MB2 | 4 | 0 | 0.0 | 1 |
| L1MD1 | 4 | 0 | 0.0 | 1 |
| L1ME3C | 4 | 0 | 0.0 | 1 |
| Lx2A1 | 4 | 0 | 0.0 | 1 |
| MER102b | 4 | 0 | 0.0 | 1 |
| MER103C | 4 | 0 | 0.0 | 1 |
| MER47B | 4 | 0 | 0.0 | 1 |
| MER63D | 4 | 0 | 0.0 | 1 |
| MER82 | 4 | 0 | 0.0 | 1 |
| MLT1E3 | 4 | 0 | 0.0 | 1 |
| MLT2B3 | 4 | 0 | 0.0 | 1 |
| MURVY-LTR | 4 | 0 | 0.0 | 1 |
| RLTR12E | 4 | 0 | 0.0 | 1 |
| RLTR13C1 | 4 | 0 | 0.0 | 1 |
| RLTR13C2 | 4 | 0 | 0.0 | 1 |
| RLTR18 | 4 | 0 | 0.0 | 1 |
| RLTR20A | 4 | 0 | 0.0 | 1 |
| RLTR20A3\_MM | 4 | 0 | 0.0 | 1 |
| RLTR24 | 4 | 0 | 0.0 | 1 |
| RLTR24B\_MM | 4 | 0 | 0.0 | 1 |
| RLTR26 | 4 | 0 | 0.0 | 1 |
| RLTR26C\_MM | 4 | 0 | 0.0 | 1 |
| RLTR30 | 4 | 0 | 0.0 | 1 |
| RLTR30E\_MM | 4 | 0 | 0.0 | 1 |
| RLTR43C | 4 | 0 | 0.0 | 1 |
| RLTR9B | 4 | 0 | 0.0 | 1 |
| RMER13A | 4 | 0 | 0.0 | 1 |
| RMER21B | 4 | 0 | 0.0 | 1 |
| RMER6D | 4 | 0 | 0.0 | 1 |
| RNERVK23-int | 4 | 0 | 0.0 | 1 |
| (TCCCC)n | 4 | 0 | 0.0 | 1 |
| U14 | 4 | 0 | 0.0 | 1 |
| BGLII\_A | 3 | 0 | 0.0 | 1 |
| (CAGCC)n | 3 | 0 | 0.0 | 1 |
| (CATATA)n | 3 | 0 | 0.0 | 1 |
| (CCCTG)n | 3 | 0 | 0.0 | 1 |
| (CGAG)n | 3 | 0 | 0.0 | 1 |
| (CGGAG)n | 3 | 0 | 0.0 | 1 |
| (CGGA)n | 3 | 0 | 0.0 | 1 |
| Charlie4a | 3 | 0 | 0.0 | 1 |
| CR1\_Mam | 3 | 0 | 0.0 | 1 |
| (CTCG)n | 3 | 0 | 0.0 | 1 |
| (CTGTG)n | 3 | 0 | 0.0 | 1 |
| ERVB4\_2-LTR\_MM | 3 | 0 | 0.0 | 1 |
| ERVB5\_1-I\_MM-int | 3 | 0 | 0.0 | 1 |
| ERVB7\_2-LTR\_MM | 3 | 0 | 0.0 | 1 |
| (GAAAA)n | 3 | 0 | 0.0 | 1 |
| (GGAA)n | 3 | 0 | 0.0 | 1 |
| (GGGTG)n | 3 | 0 | 0.0 | 1 |
| HAL1b | 3 | 0 | 0.0 | 1 |
| IAPEY\_LTR | 3 | 0 | 0.0 | 1 |
| L1MA4A | 3 | 0 | 0.0 | 1 |
| L1MA5A | 3 | 0 | 0.0 | 1 |
| L1MA9 | 3 | 0 | 0.0 | 1 |
| L1MB1 | 3 | 0 | 0.0 | 1 |
| L1MD2 | 3 | 0 | 0.0 | 1 |
| L1ME2z | 3 | 0 | 0.0 | 1 |
| LTR16C | 3 | 0 | 0.0 | 1 |
| LTR50 | 3 | 0 | 0.0 | 1 |
| LTR65 | 3 | 0 | 0.0 | 1 |
| MamSINE1 | 3 | 0 | 0.0 | 1 |
| MER102c | 3 | 0 | 0.0 | 1 |
| MER105 | 3 | 0 | 0.0 | 1 |
| MER2B | 3 | 0 | 0.0 | 1 |
| MER34A1 | 3 | 0 | 0.0 | 1 |
| MER34-int | 3 | 0 | 0.0 | 1 |
| MER44C | 3 | 0 | 0.0 | 1 |
| MER46C | 3 | 0 | 0.0 | 1 |
| MER58C | 3 | 0 | 0.0 | 1 |
| MER67C | 3 | 0 | 0.0 | 1 |
| MER99 | 3 | 0 | 0.0 | 1 |
| MLT1E2 | 3 | 0 | 0.0 | 1 |
| MLT1G3 | 3 | 0 | 0.0 | 1 |
| MLT1J1 | 3 | 0 | 0.0 | 1 |
| MLTR18D\_MM | 3 | 0 | 0.0 | 1 |
| MLTR31FA\_MM | 3 | 0 | 0.0 | 1 |
| MMAR1 | 3 | 0 | 0.0 | 1 |
| MTE2a-int | 3 | 0 | 0.0 | 1 |
| MTEa-int | 3 | 0 | 0.0 | 1 |
| MTEb-int | 3 | 0 | 0.0 | 1 |
| Plat\_L3 | 3 | 0 | 0.0 | 1 |
| RLTR12B | 3 | 0 | 0.0 | 1 |
| RLTR13A2 | 3 | 0 | 0.0 | 1 |
| RLTR13B1 | 3 | 0 | 0.0 | 1 |
| RLTR13C3 | 3 | 0 | 0.0 | 1 |
| RLTR13D3 | 3 | 0 | 0.0 | 1 |
| RLTR13D4 | 3 | 0 | 0.0 | 1 |
| RLTR16 | 3 | 0 | 0.0 | 1 |
| RLTR1D | 3 | 0 | 0.0 | 1 |
| RLTR1D2\_MM | 3 | 0 | 0.0 | 1 |
| RLTR20B3 | 3 | 0 | 0.0 | 1 |
| RLTR20B3A\_MM | 3 | 0 | 0.0 | 1 |
| RLTR20B5\_MM | 3 | 0 | 0.0 | 1 |
| RLTR31B\_Mm | 3 | 0 | 0.0 | 1 |
| RLTR31\_Mur | 3 | 0 | 0.0 | 1 |
| RLTR46A2 | 3 | 0 | 0.0 | 1 |
| RLTR6\_Mm | 3 | 0 | 0.0 | 1 |
| RLTR9A2 | 3 | 0 | 0.0 | 1 |
| RMER13A1 | 3 | 0 | 0.0 | 1 |
| RMER20A | 3 | 0 | 0.0 | 1 |
| (TTCA)n | 3 | 0 | 0.0 | 1 |
| (TTGTG)n | 3 | 0 | 0.0 | 1 |
| U1 | 3 | 0 | 0.0 | 1 |
| U17 | 3 | 0 | 0.0 | 1 |
| U2 | 3 | 0 | 0.0 | 1 |
| X7A\_LINE | 3 | 0 | 0.0 | 1 |
| Arthur1 | 2 | 0 | 0.0 | 1 |
| (ATGGTG)n | 2 | 0 | 0.0 | 1 |
| BGLII\_Mus | 2 | 0 | 0.0 | 1 |
| (CAACC)n | 2 | 0 | 0.0 | 1 |
| (CAAG)n | 2 | 0 | 0.0 | 1 |
| (CACCAT)n | 2 | 0 | 0.0 | 1 |
| (CACG)n | 2 | 0 | 0.0 | 1 |
| (CAGAG)n | 2 | 0 | 0.0 | 1 |
| (CAGG)n | 2 | 0 | 0.0 | 1 |
| (CCCG)n | 2 | 0 | 0.0 | 1 |
| (CCGAG)n | 2 | 0 | 0.0 | 1 |
| (CCGCG)n | 2 | 0 | 0.0 | 1 |
| Charlie24 | 2 | 0 | 0.0 | 1 |
| Charlie9 | 2 | 0 | 0.0 | 1 |
| (CTTTG)n | 2 | 0 | 0.0 | 1 |
| ERVB3\_1-I\_MM-int | 2 | 0 | 0.0 | 1 |
| ERVB3\_1-LTR\_MM | 2 | 0 | 0.0 | 1 |
| ERVB4\_3-I\_MM-int | 2 | 0 | 0.0 | 1 |
| ERVB7\_4-LTR\_MM | 2 | 0 | 0.0 | 1 |
| FordPrefect | 2 | 0 | 0.0 | 1 |
| (GAGAA)n | 2 | 0 | 0.0 | 1 |
| (GGAAA)n | 2 | 0 | 0.0 | 1 |
| Helitron3Na\_Mam | 2 | 0 | 0.0 | 1 |
| HERVL40-int | 2 | 0 | 0.0 | 1 |
| IAPEY2\_LTR | 2 | 0 | 0.0 | 1 |
| IAPEY3\_LTR | 2 | 0 | 0.0 | 1 |
| IAPEY5\_I-int | 2 | 0 | 0.0 | 1 |
| IAPLTR1a\_Mm | 2 | 0 | 0.0 | 1 |
| IAPLTR4 | 2 | 0 | 0.0 | 1 |
| Kanga1 | 2 | 0 | 0.0 | 1 |
| Kanga1c | 2 | 0 | 0.0 | 1 |
| Kanga2\_a | 2 | 0 | 0.0 | 1 |
| L1MB4 | 2 | 0 | 0.0 | 1 |
| L1MC2 | 2 | 0 | 0.0 | 1 |
| L1ME3B | 2 | 0 | 0.0 | 1 |
| L1ME3D | 2 | 0 | 0.0 | 1 |
| L1MEe | 2 | 0 | 0.0 | 1 |
| LTR40a | 2 | 0 | 0.0 | 1 |
| LTR41B | 2 | 0 | 0.0 | 1 |
| LTR81C | 2 | 0 | 0.0 | 1 |
| LTR82A | 2 | 0 | 0.0 | 1 |
| LTR88c | 2 | 0 | 0.0 | 1 |
| LTR89 | 2 | 0 | 0.0 | 1 |
| LTRIS4 | 2 | 0 | 0.0 | 1 |
| MamGypLTR3 | 2 | 0 | 0.0 | 1 |
| MamRep605 | 2 | 0 | 0.0 | 1 |
| MER115 | 2 | 0 | 0.0 | 1 |
| MER117 | 2 | 0 | 0.0 | 1 |
| MER31-int | 2 | 0 | 0.0 | 1 |
| MER34B-int | 2 | 0 | 0.0 | 1 |
| MER44A | 2 | 0 | 0.0 | 1 |
| MER45B | 2 | 0 | 0.0 | 1 |
| MER52-int | 2 | 0 | 0.0 | 1 |
| MER63A | 2 | 0 | 0.0 | 1 |
| MER67D | 2 | 0 | 0.0 | 1 |
| MER77 | 2 | 0 | 0.0 | 1 |
| MLT1A | 2 | 0 | 0.0 | 1 |
| MLT1E1A | 2 | 0 | 0.0 | 1 |
| MLT1F | 2 | 0 | 0.0 | 1 |
| MLT1F2 | 2 | 0 | 0.0 | 1 |
| MLT1G1 | 2 | 0 | 0.0 | 1 |
| MLT1I | 2 | 0 | 0.0 | 1 |
| MLT1-int | 2 | 0 | 0.0 | 1 |
| MLT1J2 | 2 | 0 | 0.0 | 1 |
| MLT1M | 2 | 0 | 0.0 | 1 |
| MLT2B5 | 2 | 0 | 0.0 | 1 |
| MLT2E | 2 | 0 | 0.0 | 1 |
| MLTR31A\_MM | 2 | 0 | 0.0 | 1 |
| Ricksha\_c | 2 | 0 | 0.0 | 1 |
| RLTR10B | 2 | 0 | 0.0 | 1 |
| RLTR10E | 2 | 0 | 0.0 | 1 |
| RLTR12H | 2 | 0 | 0.0 | 1 |
| RLTR13A | 2 | 0 | 0.0 | 1 |
| RLTR13D | 2 | 0 | 0.0 | 1 |
| RLTR13D1 | 2 | 0 | 0.0 | 1 |
| RLTR13D3A | 2 | 0 | 0.0 | 1 |
| RLTR13D5 | 2 | 0 | 0.0 | 1 |
| RLTR16C\_MM | 2 | 0 | 0.0 | 1 |
| RLTR17D\_Mm | 2 | 0 | 0.0 | 1 |
| RLTR19A2 | 2 | 0 | 0.0 | 1 |
| RLTR1A2\_MM | 2 | 0 | 0.0 | 1 |
| RLTR1C | 2 | 0 | 0.0 | 1 |
| RLTR20A2B\_MM | 2 | 0 | 0.0 | 1 |
| RLTR20B2 | 2 | 0 | 0.0 | 1 |
| RLTR20C | 2 | 0 | 0.0 | 1 |
| RLTR23 | 2 | 0 | 0.0 | 1 |
| RLTR27 | 2 | 0 | 0.0 | 1 |
| RLTR30D\_MM | 2 | 0 | 0.0 | 1 |
| RLTR34B\_MM | 2 | 0 | 0.0 | 1 |
| RLTR41C | 2 | 0 | 0.0 | 1 |
| RLTR44E | 2 | 0 | 0.0 | 1 |
| RLTR48B | 2 | 0 | 0.0 | 1 |
| RLTR4\_Mm | 2 | 0 | 0.0 | 1 |
| RLTR9A3A | 2 | 0 | 0.0 | 1 |
| RLTR9F | 2 | 0 | 0.0 | 1 |
| RMER17A-int | 2 | 0 | 0.0 | 1 |
| (TAAAA)n | 2 | 0 | 0.0 | 1 |
| (TCTCCC)n | 2 | 0 | 0.0 | 1 |
| (TCTCC)n | 2 | 0 | 0.0 | 1 |
| (TGAG)n | 2 | 0 | 0.0 | 1 |
| Tigger14a | 2 | 0 | 0.0 | 1 |
| (TTAGGC)n | 2 | 0 | 0.0 | 1 |
| U3 | 2 | 0 | 0.0 | 1 |
| X3\_LINE | 2 | 0 | 0.0 | 1 |
| X7B\_LINE | 2 | 0 | 0.0 | 1 |
| X9\_LINE | 2 | 0 | 0.0 | 1 |
| (AACTG)n | 1 | 0 | 0.0 | 1 |
| Arthur1B | 1 | 0 | 0.0 | 1 |
| (ATAAG)n | 1 | 0 | 0.0 | 1 |
| (ATAGG)n | 1 | 0 | 0.0 | 1 |
| (CAACG)n | 1 | 0 | 0.0 | 1 |
| (CAAT)n | 1 | 0 | 0.0 | 1 |
| (CACGC)n | 1 | 0 | 0.0 | 1 |
| (CACTG)n | 1 | 0 | 0.0 | 1 |
| (CAGAA)n | 1 | 0 | 0.0 | 1 |
| (CAGTG)n | 1 | 0 | 0.0 | 1 |
| (CAGT)n | 1 | 0 | 0.0 | 1 |
| (CATG)n | 1 | 0 | 0.0 | 1 |
| (CCCCAA)n | 1 | 0 | 0.0 | 1 |
| (CCCCCG)n | 1 | 0 | 0.0 | 1 |
| (CCCGAA)n | 1 | 0 | 0.0 | 1 |
| (CCGAA)n | 1 | 0 | 0.0 | 1 |
| (CCTAA)n | 1 | 0 | 0.0 | 1 |
| (CGAAA)n | 1 | 0 | 0.0 | 1 |
| (CGAAG)n | 1 | 0 | 0.0 | 1 |
| (CGAGG)n | 1 | 0 | 0.0 | 1 |
| (CGCGG)n | 1 | 0 | 0.0 | 1 |
| (CGCTG)n | 1 | 0 | 0.0 | 1 |
| (CGTTG)n | 1 | 0 | 0.0 | 1 |
| Charlie10 | 1 | 0 | 0.0 | 1 |
| Charlie15a | 1 | 0 | 0.0 | 1 |
| Charlie16a | 1 | 0 | 0.0 | 1 |
| Charlie18a | 1 | 0 | 0.0 | 1 |
| Charlie19a | 1 | 0 | 0.0 | 1 |
| Charlie22a | 1 | 0 | 0.0 | 1 |
| Charlie25 | 1 | 0 | 0.0 | 1 |
| Charlie26a | 1 | 0 | 0.0 | 1 |
| Charlie7 | 1 | 0 | 0.0 | 1 |
| Charlie7a | 1 | 0 | 0.0 | 1 |
| (C)n | 1 | 0 | 0.0 | 1 |
| (CTAAA)n | 1 | 0 | 0.0 | 1 |
| (CTA)n | 1 | 0 | 0.0 | 1 |
| ERV3-16A3\_I-int | 1 | 0 | 0.0 | 1 |
| ERVB4\_1-LTR\_MM | 1 | 0 | 0.0 | 1 |
| ERVB4\_3-LTR\_MM | 1 | 0 | 0.0 | 1 |
| ERVB5\_2-LTR\_MM | 1 | 0 | 0.0 | 1 |
| ERVB7\_3-LTR\_MM | 1 | 0 | 0.0 | 1 |
| Eulor2B | 1 | 0 | 0.0 | 1 |
| Eulor5A | 1 | 0 | 0.0 | 1 |
| Eulor6B | 1 | 0 | 0.0 | 1 |
| FAM | 1 | 0 | 0.0 | 1 |
| (GCCTAA)n | 1 | 0 | 0.0 | 1 |
| (GGAGA)n | 1 | 0 | 0.0 | 1 |
| (GGGAGA)n | 1 | 0 | 0.0 | 1 |
| (GTCTG)n | 1 | 0 | 0.0 | 1 |
| HY1 | 1 | 0 | 0.0 | 1 |
| IAPEY4\_LTR | 1 | 0 | 0.0 | 1 |
| Kanga11a | 1 | 0 | 0.0 | 1 |
| Kanga1b | 1 | 0 | 0.0 | 1 |
| L1M2b | 1 | 0 | 0.0 | 1 |
| L1M7 | 1 | 0 | 0.0 | 1 |
| L1MA10 | 1 | 0 | 0.0 | 1 |
| L1MD3 | 1 | 0 | 0.0 | 1 |
| L1MDb | 1 | 0 | 0.0 | 1 |
| L1ME3 | 1 | 0 | 0.0 | 1 |
| L1ME3A | 1 | 0 | 0.0 | 1 |
| L1ME3F | 1 | 0 | 0.0 | 1 |
| L1MEd | 1 | 0 | 0.0 | 1 |
| L1MEg1 | 1 | 0 | 0.0 | 1 |
| Looper | 1 | 0 | 0.0 | 1 |
| LTR16A1 | 1 | 0 | 0.0 | 1 |
| LTR16A2 | 1 | 0 | 0.0 | 1 |
| LTR16B | 1 | 0 | 0.0 | 1 |
| LTR16B1 | 1 | 0 | 0.0 | 1 |
| LTR16D1 | 1 | 0 | 0.0 | 1 |
| LTR16D2 | 1 | 0 | 0.0 | 1 |
| LTR31 | 1 | 0 | 0.0 | 1 |
| LTR33 | 1 | 0 | 0.0 | 1 |
| LTR33A | 1 | 0 | 0.0 | 1 |
| LTR33C | 1 | 0 | 0.0 | 1 |
| LTR40b | 1 | 0 | 0.0 | 1 |
| LTR41 | 1 | 0 | 0.0 | 1 |
| LTR45 | 1 | 0 | 0.0 | 1 |
| LTR48 | 1 | 0 | 0.0 | 1 |
| LTR53B | 1 | 0 | 0.0 | 1 |
| LTR75 | 1 | 0 | 0.0 | 1 |
| LTR78B | 1 | 0 | 0.0 | 1 |
| LTR79 | 1 | 0 | 0.0 | 1 |
| LTR81B | 1 | 0 | 0.0 | 1 |
| LTR84b | 1 | 0 | 0.0 | 1 |
| LTR85a | 1 | 0 | 0.0 | 1 |
| LTR85c | 1 | 0 | 0.0 | 1 |
| MADE2 | 1 | 0 | 0.0 | 1 |
| MamGypLTR1b | 1 | 0 | 0.0 | 1 |
| MamGypLTR1d | 1 | 0 | 0.0 | 1 |
| MamRep1161 | 1 | 0 | 0.0 | 1 |
| MamRep137 | 1 | 0 | 0.0 | 1 |
| MamRep38 | 1 | 0 | 0.0 | 1 |
| MamRep564 | 1 | 0 | 0.0 | 1 |
| MER102a | 1 | 0 | 0.0 | 1 |
| MER104 | 1 | 0 | 0.0 | 1 |
| MER106A | 1 | 0 | 0.0 | 1 |
| MER124 | 1 | 0 | 0.0 | 1 |
| MER130 | 1 | 0 | 0.0 | 1 |
| MER131 | 1 | 0 | 0.0 | 1 |
| MER31A | 1 | 0 | 0.0 | 1 |
| MER31B | 1 | 0 | 0.0 | 1 |
| MER34 | 1 | 0 | 0.0 | 1 |
| MER34A | 1 | 0 | 0.0 | 1 |
| MER44D | 1 | 0 | 0.0 | 1 |
| MER45A | 1 | 0 | 0.0 | 1 |
| MER50B | 1 | 0 | 0.0 | 1 |
| MER54A | 1 | 0 | 0.0 | 1 |
| MER54B | 1 | 0 | 0.0 | 1 |
| MER57E2 | 1 | 0 | 0.0 | 1 |
| MER58D | 1 | 0 | 0.0 | 1 |
| MER63B | 1 | 0 | 0.0 | 1 |
| MER63C | 1 | 0 | 0.0 | 1 |
| MER68 | 1 | 0 | 0.0 | 1 |
| MER68B | 1 | 0 | 0.0 | 1 |
| MER70B | 1 | 0 | 0.0 | 1 |
| MER77B | 1 | 0 | 0.0 | 1 |
| MER88 | 1 | 0 | 0.0 | 1 |
| MER91B | 1 | 0 | 0.0 | 1 |
| MER92B | 1 | 0 | 0.0 | 1 |
| MER94 | 1 | 0 | 0.0 | 1 |
| MER96B | 1 | 0 | 0.0 | 1 |
| MLT1A0-int | 1 | 0 | 0.0 | 1 |
| MLT1A1-int | 1 | 0 | 0.0 | 1 |
| MLT1D-int | 1 | 0 | 0.0 | 1 |
| MLT1E | 1 | 0 | 0.0 | 1 |
| MLT1F1 | 1 | 0 | 0.0 | 1 |
| MLT1G | 1 | 0 | 0.0 | 1 |
| MLT1H | 1 | 0 | 0.0 | 1 |
| MLT1H1 | 1 | 0 | 0.0 | 1 |
| MLT1H2 | 1 | 0 | 0.0 | 1 |
| MLT1J1-int | 1 | 0 | 0.0 | 1 |
| MLT1N2 | 1 | 0 | 0.0 | 1 |
| MLT2B2 | 1 | 0 | 0.0 | 1 |
| MLT2B4 | 1 | 0 | 0.0 | 1 |
| MLT2C2 | 1 | 0 | 0.0 | 1 |
| MLT-int | 1 | 0 | 0.0 | 1 |
| MLTR18A\_MM | 1 | 0 | 0.0 | 1 |
| MLTR18C\_MM | 1 | 0 | 0.0 | 1 |
| MLTR18\_MM | 1 | 0 | 0.0 | 1 |
| MLTR25C | 1 | 0 | 0.0 | 1 |
| MLTR31E\_MM | 1 | 0 | 0.0 | 1 |
| MMTV-int | 1 | 0 | 0.0 | 1 |
| Ricksha | 1 | 0 | 0.0 | 1 |
| Ricksha\_0 | 1 | 0 | 0.0 | 1 |
| RLTR10A | 1 | 0 | 0.0 | 1 |
| RLTR10D2 | 1 | 0 | 0.0 | 1 |
| RLTR10F | 1 | 0 | 0.0 | 1 |
| RLTR12A | 1 | 0 | 0.0 | 1 |
| RLTR12G | 1 | 0 | 0.0 | 1 |
| RLTR13B4 | 1 | 0 | 0.0 | 1 |
| RLTR13F | 1 | 0 | 0.0 | 1 |
| RLTR19A | 1 | 0 | 0.0 | 1 |
| RLTR19B | 1 | 0 | 0.0 | 1 |
| RLTR19C | 1 | 0 | 0.0 | 1 |
| RLTR20A2 | 1 | 0 | 0.0 | 1 |
| RLTR20D | 1 | 0 | 0.0 | 1 |
| RLTR28B | 1 | 0 | 0.0 | 1 |
| RLTR31A\_Mm | 1 | 0 | 0.0 | 1 |
| RLTR31\_Mm | 1 | 0 | 0.0 | 1 |
| RLTR34C\_MM | 1 | 0 | 0.0 | 1 |
| RLTR35B\_MM | 1 | 0 | 0.0 | 1 |
| RLTR44B | 1 | 0 | 0.0 | 1 |
| RLTR46 | 1 | 0 | 0.0 | 1 |
| RLTR46A | 1 | 0 | 0.0 | 1 |
| RLTR47 | 1 | 0 | 0.0 | 1 |
| RLTR48C | 1 | 0 | 0.0 | 1 |
| RLTR5\_Mm | 1 | 0 | 0.0 | 1 |
| RLTR6C\_Mm | 1 | 0 | 0.0 | 1 |
| RLTR8 | 1 | 0 | 0.0 | 1 |
| RLTR9A3 | 1 | 0 | 0.0 | 1 |
| RLTR9B2 | 1 | 0 | 0.0 | 1 |
| RLTR9C | 1 | 0 | 0.0 | 1 |
| RLTR9D2 | 1 | 0 | 0.0 | 1 |
| RMER13A2 | 1 | 0 | 0.0 | 1 |
| (TAAAAA)n | 1 | 0 | 0.0 | 1 |
| (TAG)n | 1 | 0 | 0.0 | 1 |
| (TCCCG)n | 1 | 0 | 0.0 | 1 |
| (TCCG)n | 1 | 0 | 0.0 | 1 |
| (TCG)n | 1 | 0 | 0.0 | 1 |
| (TGGG)n | 1 | 0 | 0.0 | 1 |
| Tigger12c | 1 | 0 | 0.0 | 1 |
| Tigger16a | 1 | 0 | 0.0 | 1 |
| Tigger1a\_Art | 1 | 0 | 0.0 | 1 |
| Tigger2b | 1 | 0 | 0.0 | 1 |
| Tigger8 | 1 | 0 | 0.0 | 1 |
| Tigger9b | 1 | 0 | 0.0 | 1 |
| tRNA-Ala-GCG | 1 | 0 | 0.0 | 1 |
| tRNA-Gln-CAA\_ | 1 | 0 | 0.0 | 1 |
| tRNA-Gly-GGA | 1 | 0 | 0.0 | 1 |
| tRNA-Leu-TTA(m) | 1 | 0 | 0.0 | 1 |
| (TTAA)n | 1 | 0 | 0.0 | 1 |
| (TTAGGG)n | 1 | 0 | 0.0 | 1 |
| (TTTAA)n | 1 | 0 | 0.0 | 1 |
| (TTTAG)n | 1 | 0 | 0.0 | 1 |
| U13\_ | 1 | 0 | 0.0 | 1 |
| U8 | 1 | 0 | 0.0 | 1 |
| UCON2 | 1 | 0 | 0.0 | 1 |
| UCON23 | 1 | 0 | 0.0 | 1 |
| UCON29 | 1 | 0 | 0.0 | 1 |
| UCON4 | 1 | 0 | 0.0 | 1 |
| X2\_LINE | 1 | 0 | 0.0 | 1 |
| X5B\_LINE | 1 | 0 | 0.0 | 1 |
| X6A\_LINE | 1 | 0 | 0.0 | 1 |
| X6B\_LINE | 1 | 0 | 0.0 | 1 |
| X7C\_LINE | 1 | 0 | 0.0 | 1 |
| X8\_LINE | 1 | 0 | 0.0 | 1 |
| Zaphod | 1 | 0 | 0.0 | 1 |
| Zaphod2 | 1 | 0 | 0.0 | 1 |
